# Increased Carotid Intima-Media Thickness Associated with Antibody Responses to Varicella-Zoster Virus and Cytomegalovirus in HIV-Infected Patients

**DOI:** 10.1371/journal.pone.0064327

**Published:** 2013-05-23

**Authors:** Mar Masiá, Catalina Robledano, Victoria Ortiz de la Tabla, Pedro Antequera, Natividad López, Félix Gutiérrez

**Affiliations:** 1 Infectious Diseases Unit, Hospital General Universitario de Elche, Universidad Miguel Hernández, Alicante, Spain; 2 Microbiology Service, Hospital Universitario de San Juan, Alicante, Spain; 3 Biochemistry Section, Hospital General Universitario de Elche, Elche, Alicante, Spain; University of Hawaii Manoa, United States of America

## Abstract

**Objective:**

We investigated the relationship of the *Herpesviridiae* with inflammation and subclinical atherosclerosis in HIV-infected patients.

**Methods:**

Prospective study including virologically suppressed HIV-infected patients. IgG antibodies against herpesviruses, carotid intima-media thickness (cIMT), endothelial function through flow-mediated dilatation (FMD) of the brachial artery, and blood atherosclerosis biomarkers (hsCRP, TNF-α, IL-6, MCP-1, MDA, sCD14, sCD163, VCAM-1, ICAM-1, D-dimer, and PAI-1) were measured.

**Results:**

136 patients with HIV viral load <200 copies/ml were included. 93.4% patients were infected with herpes simplex virus type-1, 55.9% with herpes simplex virus type-2, 97.1% with varicella-zoster virus, 65.4% with human herpesvirus-6, 91.2% with cytomegalovirus, and 99.3% with Epstein-Barr virus. Previous AIDS diagnosis was associated with higher cytomegalovirus IgG titers (23,000 vs 17,000 AU, P = 0.011) and higher varicella-zoster virus IgG titers (3.19 vs 2.88 AU, P = 0.047), and there was a positive correlation of the Framingham risk score with IgG levels against cytomegalovirus (Spearman's Rho 0.216, P = 0.016) and Herpes simplex virus-2 (Spearman's Rho 0.293, P = 0.001). IgG antibodies against cytomegalovirus correlated in adjusted analysis with the cIMT (P = 0.030). High seropositivity for varicella-zoster virus (OR 2.91, 95% CI 1.05–8.01, P = 0.039), and for cytomegalovirus (OR 3.79, 95% CI 1.20–11.97, P = 0.023) were predictors for the highest quartile of the cIMT in adjusted analyses. PAI-1 levels were independently associated with cytomegalovirus IgG titers (P = 0.041), IL-6 and ICAM-1 levels with varicella-zoster virus IgG (P = 0.046 and P = 0.035 respectively), and hsCRP levels with Herpes simplex virus-2 IgG (P = 0.035).

**Conclusion:**

In virologically suppressed HIV-infected patients, antibody responses against herpesviruses are associated with subclinical atherosclerosis, and with increased inflammation and coagulation biomarkers.

## Introduction

Cardiovascular disease has emerged as an important cause of morbidity and mortality in HIV-infected patients. Among the involved causes, in recent years systemic inflammation and immune activation have gained attention as central factors in the pathogenesis of HIV-related atherosclerosis [1]. While HIV replication has been considered the major trigger of the immune system, persistent inflammation and immune activation have also been found in patients receiving effective antiretroviral therapy (ART) [2], and in elite controllers [3]. This suggests that additional causes may elicit immune activation and might therefore be involved in the accelerated course of atherosclerotic disease in virologically controlled HIV-infected patients [3].

There is accumulating evidence that certain infectious agents, like *Herpesviridiae*, play a role in the pathogenesis of atherosclerosis [4]–[6]. In HIV-infected patients, a relationship with subclinical atherosclerosis has been reported for cytomegalovirus and herpes simplex virus type-2 (HSV-2) [7], [8], [9]. To assess the role of further members of the herpesvirus family, we investigated their relationship with carotid intima-media thickness (cIMT), endothelial function through flow-mediated dilatation (FMD) of the brachial artery, and several cardiovascular biomarkers in virologically suppressed HIV-infected patients.

## Methods

We conducted a prospective study including consecutive HIV-infected patients at increased risk for herpesvirus infection cared for in an HIV outpatient clinic. Accordingly, patients who acquired the HIV through sexual transmission were invited to participate in the study. To avoid the confounding effect of HIV replication, only patients with virological suppression, defined as a viral load <200 copies/ml, were included in the study.

### Ethics statement

Patients who agreed signed an informed consent before inclusion. The local ethics committee (Comité Ético del Hospital General Universitario de Elche) approved the protocol. The study protocol was in accordance with the Declaration of Helsinki of good clinical practice guidelines

### Clinical and laboratory measurements

Details were taken of HIV-related data, cardiovascular risk factors, and hepatitis C (HCV) and B virus (HBV) coinfection. Blood tests, c-IMT, and FMD of the brachial artery were obtained after at least an 8 hour overnight fast and smoking abstinence. Plasma aliquots obtained were stored at −80°C. All frozen samples were subsequently defrosted, and levels of vascular cell adhesion molecule-1 (VCAM-1), intercellular CAM-1 (ICAM-1), monocyte chemoattractant protein-1 (MCP-1), interleukin-6 (IL-6), tumour necrosis factor-alpha (TNF-α), plasminogen activator inhibitor (PAI-1), sCD14 and sCD163 were measured using commercially available ELISA kits (Quantikine, R&D Systems Europe Ltd, UK). Highly-sensitive C-reactive protein (hsCRP) was measured with a chemiluminescent immunometric assay (Immwulite 2000, Siemens) as previously described [10]. D-dimer was measured with TECHNOZYM® D-Dimer ELISA, TECHNOCLONE GMBH (Vienna, Austria), and malondialdehyde (MDA) with HPLC analysis (CHROMSYSTEMS, München-Germany).

IgG antibodies against several herpesviruses were measured by commercial ELISA test kits: FOCUS Diagnostics (Cypress, CA. U.S.A) for HSV-1 and HSV-2; Trinity Biotech (Co Wicklow, Ireland) for Epstein-Barr virus (EBV); Vircell SL (Granada, Spain) for varicella-zoster virus; Panbio Ltd. Inverness Medical Innovations (Sinnamon Park, Australia) for human herpesvirus type 6 (HHV-6), and Siemens Healthcare Diagnostics (Barcelona, Spain) for CMV. Results are provided in absorbance units (AU). The recommended cut-off point of >1.1 was considered positive, except for CMV (>230 AU).

### Evaluation of the endothelial function and the carotid intima-media thickness

Endothelial function was evaluated by measuring FMD of the brachial artery as detailed elsewhere [11]. Measurements were performed by an experienced examiner blinded to the subject's information. Reproducibility was assessed by the same examiner in 10 healthy subjects (8 men, age 44±8.5 years) who had FMD measured twice, at an interval of 2 hours. Median (interquartile range [IQR]) FMD was 8.15% (5.26–12.1%). Median (IQR) intraobserver intersession percentage of variation for brachial artery diameter was 2.38% (0–5.71), within the reported range. cIMT measurement was performed as previously described [12]. Measures were taken from both common carotids and bulb portions. Total cIMT was calculated as the mean of all measurements, and analyzed both as a continuous variable, and categorized in quartiles.

### Statistical analyses

Bivariate Spearman's correlation and Kruskal-Wallis tests were used to assess the association of IgG antibodies against the herpesviruses with the cIMT, FMD and blood biomarkers. Multivariable linear regression and logistic regression analyses were performed to adjust the models for the variables found to be associated in univariate analyses: Framingham risk score, HCV, and previous AIDS diagnosis. To be included in regression analyses, all continuous variables were transformed on the natural log scale (log_e_) due to their highly skewed distribution.

## Results

157 patients on ART were screened; of them, 140 had an HIV viral load <200 copies/ml, and in 136 serological results were available. Median (IQR) age was 47 (41–54) years, and 139 were men. Median (IQR) CD4 cell count was 650 (437–862) cell/mm3, 40 (29.4%) had been previously diagnosed of AIDS, 14 (10%) patients were co-infected with HCV, and 10 (7.1%) with HBV. The most frequent antiretroviral regimens contained protease inhibitors (43.4%), and non nucleoside reverse transcriptase inhibitors (39%). A total of 49.3% patients were smokers, 46.4% had hyperlipidemia, 27.9% hypertension,7.1% were diabetic, 33.1% were receiving lipid-lowering therapy, and 19.9% antihypertensive agents. Median (IQR) Framingham score was 6.5% (2–12%). Framingham risk score correlated with IgG antibody levels against cytomegalovirus (Spearman's rho 0.216, P = 0.016) and with HSV-2 IgG levels (Spearman's rho 0.293, P = 0.001).

Quantitative and qualitative serology tests results are shown in Table 1. Previous AIDS diagnosis was associated with higher IgG anti-cytomegalovirus titers (23,000 vs 17,000 AU, P = 0.011) and higher IgG anti- varicella-zoster virus titers (3.19 vs 2.88 AU, P = 0.047). There was a positive correlation of the Framingham risk score with IgG levels against cytomegalovirus and HSV-2 (Table 2). Hepatitis C was associated with lower IgG titers against cytomegalovirus (13,000 vs 19,000 AU, P = 0.047), HHV-6 (1.21 vs 1.76 AU, P = 0.019) and a trend to lower titers against HSV-2 (0.27 vs 8.61 AU, P = 0.058). No differences in IgG levels were found according to hepatitis B serostatus or antiretroviral therapy composition. No correlation was found between CD4 cells and antibody levels against any of the herpesviruses (data not shown).

**Table 1:** Baseline characteristics of the patients.

Variable	All patients(N = 136)
Age, years, mean (SD)	48.6 (11.6)
Sex, women	1 (0.7)
Race, Caucasian	132 (97.1)
Previous AIDS diagnosis	40 (29.4)
CD4 cell count, cell/mm^3^	650 (437–862)
PI-including regimen	59 (43.4)
NNRTI-including regimen	53 (39.0)
Framingham risk score, %	6.5 (2.0–12.0)
Total cIMT, mm	0.78 (0.65–0.98)
Left bulb cIMT, mm	0.85 (0.70–1.14)
Right bulb cIMT, mm	0.90 (0.70–1.20)
Carotid plaques (IMT>1.5)	33 (23.57)
Flow-mediated dilatation, %	4.04 (1.03–8.38)
hsCRP, mg/L	2.51 (1.06–4.74)
IL-6, pg/mL	2.73 (0.88–7.24)
TNF-α, pg/mL	19.7 (7.0–35.6)
sICAM-1, ng/mL	64.8 (18.0–212.1)
sVCAM-1, ng/mL	450.9 (143.2–739.0)
MCP-1, pg/mL	687.8 (177.7–1374.4)
CD4/CD38/HLA-DR, %	4.51 (2.75–10.43)
CD8/CD38/HLA-DR, %	5.43 (3.70–9.39)
PAI-1, ng/mL	13.25 (3.65–25.46)
D-dimer, ng/mL	46.33 (25.23–68.01)
MDA, µmol/L	0.13 (0.10–0.15)
sCD14, pg/mL	5,5546 (3,196–13,834)
sCD163, ng/mL	942.5 (638.5–1,268)
Hepatitis C coinfection	14 (10)
Hepatitis B coinfection	10 (7.1)
Herpes simplex virus type-1, no. (%)	127 (93.4)
Median IgG levels (IQR), AU	7.60 (4.56–9.82)
Herpes simplex virus type-2, no. (%)	76 (55.9)
Median IgG levels (IQR), AU	3.20 (0.16–8.48)
Varicella-zoster virus, no. (%)	132 (97.1)
Median IgG levels (IQR), AU	2.97 (2.45–3.34)
Human herpesvirus-6, no. (%)	89 (65.4)
Median IgG levels (IQR), AU	1.28 (0.95–1.73)
Cytomegalovirus, no. (%)	124 (91.2)
Median IgG levels (IQR), AU	19,000 (13,000–29,000)
Epstein-Barr virus, no. (%)	135 (99.3)
Median IgG levels (IQR), AU	6.02 (5.37–7.01)

Continuous variables are expressed in median (interquartile range), unless indicated. Categorical variables are expressed in no. (%).

cIMT, carotid intima-media thickness; FMD, flow-mediated dilatation; hsCRP, highly-sensitive C-reactive protein; VCAM-1, vascular cell adhesion molecule-1; ICAM-1, intercellular cell adhesion molecule-1; IL-6, interleukin-6; MDA, malondialdehyde; TNF-α, tumour necrosis factor-alpha; PAI-1, plasminogen activator inhibitor-1; MCP-1, monocyte chemoattractant protein-1; HSV-1, herpes simplex virus type-1; VZV, varicella zoster virus; HHV-6, human herpesvirus 6; EBV, Epstein-Barr virus; CMV, cytomegalovirus; IQR, interquartile range; AU, absorbance units.

**Table 2:** Correlation between the levels of biomarkers and IgG antibodies against several herpesviruses.

	HSV-1		HVS-2		VZV		HHV-6		EBV		CMV	
	Spearman's rho	P	Spearman's rho	P	Spearman's rho	P	Spearman's rho	P	Spearman's rho	P	Spearman's rho	P
Framingham score	0.120	0.17	0.293	0.001	0.061	0.488	−0.21	0.81	0.101	0.27	0.216	0.016
cIMT	0.104	0.266	0.169	0.071	0.169	0.07	−0.141	0.131	0.00	0.998	0.273	0.004
FMD	−0.030	0.739	0.121	0.179	0.097	0.284	0.121	0.179	−0.169	0.062	0.003	0.974
hsCRP	0.099	0.251	0.263	0.002	−0.061	0.477	0.113	0.192	−0.013	0.885	0.192	0.030
VCAM-1	−0.095	0.270	0.095	0.269	0.059	0.494	−0.026	0.761	0.068	0.440	0.81	0.363
ICAM-1	0,033	0.699	0.012	0.894	0.330	0.000	−0.088	0.308	−0.319	0.000	0.065	0.466
IL-6	0.059	0.494	−0.013	0.884	0.314	0.000	−0.063	0.468	−0.277	0.001	0.028	0.755
MDA	0,171	0.047	−0.57	0.509	0.094	0.275	−0.092	0.286	0.050	0.567	0.064	0.476
TNF-a	−0.071	0.414	0.046	0.595	0.057	0.506	0.057	0.513	0.059	0.500	0.055	0.540
PAI-1	0.081	0.354	−0.028	0.748	0.101	0.246	0.106	0.224	−0.036	0.681	0.182	0.042
MCP-1	−0.104	0.229	0.082	0.344	0.012	0.888	0.069	0.428	0.127	0.147	0.048	0.589
D-dimer	−0.002	0.981	−0.017	0.847	0.007	0.935	−0.082	0.345	0.002	0.982	−0.146	0.102
sCD14	0.115	0.181	−0.010	0.908	0.108	0.212	0.061	0.478	−0.111	0.203	0.057	0.522
sCD163	0.018	0.838	0.023	0.795	−0.058	0.511	−0.093	0.292	0.051	0.562	0.006	0.950

cIMT, carotid intima-media thickness; FMD, flow-mediated dilatation; hsCRP, highly-sensitive C-reactive protein; VCAM-1, vascular cell adhesion molecule-1; ICAM-1, intercellular cell adhesion molecule-1; IL-6, interleukin-6; MDA, malondialdehyde; TNF-α, tumour necrosis factor-alpha; PAI-1, plasminogen activator inhibitor-1; MCP-1, monocyte chemoattractant protein-1; HSV-1, herpes simplex virus type 1; VZV, varicella zoster virus; HHV-6, human herpesvirus 6; EBV, Epstein-Barr virus; CMV, cytomegalovirus.

### Carotid intima-media thickness

There was a significant positive correlation between total cIMT and IgG antibodies against cytomegalovirus (P = 0.004), and a trend to a correlation with varicella-zoster virus (P = 0.070) and HSV-2 (P = 0.071) IgG levels (Table 2). IgG cytomegalovirus antibodies also correlated with the cIMT at all locations measured: right common cIMT (Spearman's rho 0.241, P = 0.008), left common cIMT (Spearman's rho 0.197, P = 0.031), right bulb (Spearman's rho 0.199, P = 0.034), and left bulb (Spearman's rho 0.236, P = 0.012). VZV IgG antibodies correlated with the right bulb cIMT (Spearman's rho, 0.198, P = 0.029). Linear regression analyses adjusted for the Framingham score, HCV, and previous AIDS diagnosis, showed a persistent association of IgG cytomegalovirus antibodies with total cIMT (P = 0.030) and with the cIMT at different locations (P = 0.027 and P = 0.012 for left bulb and right common cIMT, and P = 0.087 for left common cIMT).

**Figure 1:**
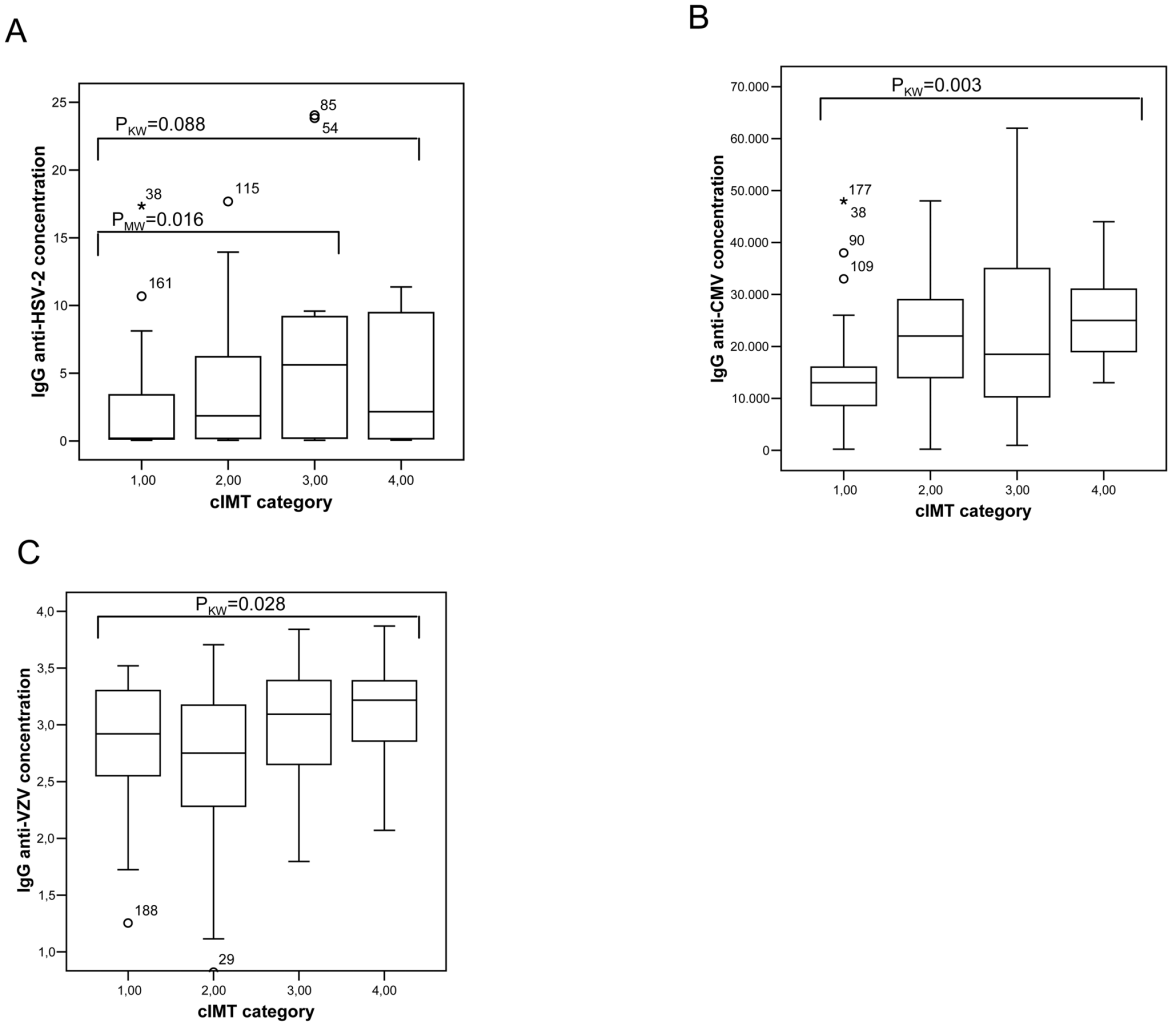
Relationship of IgG antibody levels against HSV-2, CMV and VZV with cIMT quartiles. cIMT categories correspond to total cIMT quartiles, calculated as the media of the sum of all cIMT measurements and divided as follows: 1^st^ quartile (category 1): 0.49–0.65 mm; 2^nd^ quartile (category 2): ≥0.65–0.78 mm; 3^rd^ quartile (category 3): ≥0.78–0.98 mm; 4^th^ quartile (category 4): ≥0.98–1.80 mm.

When total cIMT was categorized in quartiles, a significant increase of IgG antibodies against cytomegalovirus (P_KW_ = 0.003), and against varicella-zoster virus (P_KW_ 0.028) were found with increasing total cIMT quartiles (Figure 1). For HSV-2, there was a significant difference in IgG antibody levels between the 1^st^ and 3^rd^ total cIMT quartiles (P = 0.016). No other significant associations were found for the other herpesviruses analyzed. The highest cIMT quartile (≥0.98 mm) was selected to define subclinical atherosclerosis, and IgG antibody levels above the median to define high seropositivity. In multivariate logistic regression analyses adjusted for the Framingham score, HCV, and previous AIDS diagnosis, subclinical atherosclerosis was independently associated with high varicella-zoster virus seropositivity (≥2.97 AU) (OR 2.91, 95% CI 1.05–8.01, P = 0.039), and with high cytomegalovirus seropositivity (≥19,000 AU) (OR 3.79, 95% CI 1.20–11.97, P = 0.023) (Table 3). The association between subclinical atherosclerosis and high varicella-zoster seropositivity remained significant when IgG antibodies against cytomegalovirus were included in the model (OR 3.05; 95% CI, 1.05–8.87; P = 0.041). Likewise, the association of subclinical atherosclerosis with high cytomegalovirus seropositivity remained significant when IgG antibodies against varicella-zoster virus were included in the model (OR 3.89; 95% CI, 1.12–13.52; P = 0.033). In addition, when the models were adjusted for each of the traditional cardiovascular risk factors not influenced by treatment (age, ethnicity, smoking and diabetes) instead of the Framingham score, and also for the antiretroviral regimen composition (protease inhibitors vs non nucleoside reverse transcriptase inhibitors), high seropositivity for varicella-zoster virus remained associated with carotid atherosclerosis (OR [95% CI] 5.27 [1.55–17.95], P = 0.008), and high seropositivity for cytomegalovirus was very close to significance (OR [95% CI] 3.27 [0.95–11.20], P = 0.06).

**Table 3:** Adjusted^1^ logistic regression models for the association of the highest cIMT quartile with varicella-zoster virus and cytomegalovirus IgG levels.

4^th^ cIMT quartile (0.98–1.68 mm)	OR	95% CI	P
High VZV IgG levels^2^	2.91	1.05–8.01	0.039
High VZV IgG levels+IL-6	3.20	1.06–9.62	0.039
High VZV IgG levels+ICAM-1	3.09	1.04–9.23	0.043
High CMV IgG levels^3^	3.79	1.20–11.97	0.023
High CMV IgG levels+hsCRP	4.43	1.34–14.69	0.015
High CMV IgG levels+PAI-1	3.67	1.15–11.70	0.028

1 Adjusted for the Framingham risk score, hepatitis C virus, and previous AIDS diagnosis.

cIMT, carotid intima-media thickness; VZV, varicella-zoster virus; CMV, cytomegalovirus; OR, odds ratio; CI, confidence interval; AU, absorbance units; IL-6, interleukin-6; ICAM-1, intercellular cell adhesion molecule-1; hsCRP, high sensitivity C-reactive protein; PAI-1, plasminogen activator inhibitor-1.

2 ≥2.967 AU.

3 >19,000 AU.

Further adjustment of the models shown in Table 3 for the biomarkers associated with IgG antiviral responses against cytomegalovirus or varicella-zoster virus in univariate or multivariate analyses, showed a small effect size in the association between the cIMT and the antibody responses (Table 3).

IgG antibody titers against herpesviruses tended to be higher in patients with carotid plaques, except for HHV-6, but the difference only reached statistical significance for cytomegalovirus: median (IQR) IgG 17,000 (11,325–33,500) AU in patients with plaques vs 14,000 (3115–19,500) AU in patients without plaques, P = 0.007.

### Plasma biomarkers

hsCRP levels correlated with HSV-2 (P = 0.002) and cytomegalovirus (P = 0.03) antibody titers (Table 2). After adjusted linear regression, only the association between hsCRP levels and HSV-2 antibody titers continued to be significant (P = 0.035). The biomarkers IL-6 and ICAM-1 correlated with IgG antibodies against varicella-zoster virus (P<0.001 for both), and this correlation was confirmed after adjustment (P = 0.046 and P = 0.035 respectively). Cytomegalovirus IgG antibody levels also correlated with PAI-1 in unadjusted (Spearman's rho 0.182, P = 0.042) and adjusted analyses (P = 0.041).

Total cIMT and any individual cIMT measurement correlated with sCD163 (Spearman's rho 0.28, P = 0.002 for total cIMT). Carotid bulb IMT correlated with hsCRP (Spearman's rho 0.21, P = 0.02), MCP-1 (Spearman's rho 0.22, P = 0.014), VCAM-1 (Spearman's rho 0.22, P = 0.016), and TNF-α (Spearman's rho 0.23, P = 0.011).

### Endothelial function

No statistically significant correlations were found between FMD of the brachial artery values and IgG antibody levels for any of the herpesviruses studied.

## Discussion

We explored the relationship of several *Herpesviridiae* with subclinical atherosclerosis measured with the cIMT and FMD, and different biomarkers of inflammation, endothelial activation, coagulation, and oxidative stress in HIV-infected patients. To date, no such extensive *Herpesviridiae* evaluation had been carried out to investigate their association with so many different surrogate markers of atherosclerosis in virologically-suppressed patients. Our study shows a significant and robust association between IgG antibody response against cytomegalovirus and cIMT measurements. A high seropositivity for varicella-zoster virus and for cytomegalovirus were associated with subclinical atherosclerosis. Infection with those viruses was also accompanied by a concomitant elevation of inflammation and coagulation biomarkers. Finally, anti-HSV-2 titers were associated with inflammation measured with hsCRP, but the relationship with cIMT was not confirmed in adjusted analysis.

Cellular specific anti-cytomegalovirus response had been found to be associated with the cIMT in HIV-infected patients [7]. Our results show that, in addition, a high humoral anti-cytomegalovirus response, which might reflect either an increased inflammatory individual response or the persistence of a higher load of the virus, is a predictor of increased cIMT. The higher cytomegalovirus antibody levels in patients with carotid plaques reinforce its relationship with carotid atherosclerosis. Findings from this study support those described in the general population [13], [14]. In HIV-infected patients, cytomegalovirus antibody titers have been related to decreased artery distensibility and carotid lesions in women, but no association was found with the cIMT measurements [8]. Apart from sex, the majority of African American/black and of viremic women in that study could have contributed to explain differences with ours. Interestingly, cytomegalovirus serologic response was accompanied by elevated PAI-1 levels. In vitro data support a loss of anticoagulant and the acquisition of procoagulant properties in endothelial cells infected with herpesviruses, including cytomegalovirus, which can also indirectly induce a prothrombotic state by increasing binding sites for inflammatory cells [5].

Varicella-zoster virus IgG antibodies were not as firmly correlated with the cIMT as cytomegalovirus antibodies were. However, a high serological response to varicella-zoster virus was associated with subclinical atherosclerosis. To the best of our knowledge, the relationship of this prevalent herpesvirus with atherosclerosis had not been previously described in HIV or in immunocompetent patients. This association remained after adjusting for serological response to cytomegalovirus, suggesting that it was independent from cytomegalovirus immune effects, though there may be numerous common mechanisms involved in atherosclerosis interacting each other. Additional data corroborating this finding are warranted, as well as the elevation of IL-6 and ICAM-1 accompanying varicella-zoster virus serological response, and their potential contributing role in atherogenesis. The small effect size of these two biomarkers in the model for the association of varicella-zoster virus with the cIMT, and also of the biomarkers associated with cytomegalovirus, reflects the multiple pathways contributing to atherogenesis, and probably that the antibody response to the viruses comprises several of such pro-atherogenic mechanisms, and therefore the association with atherosclerosis may be stronger than that of any biomarker alone.

No independent relationship between seropositivity to HSV-2 and the cIMT was demonstrated in our study, although higher coronary calcium levels have been described in HSV-2-infected patients [9]. Previous studies in HIV-negative patients could neither demonstrate this relationship [14]. Nevertheless, HSV-2 seropositivity was associated with hsCRP levels, and therefore its contribution to atherogenesis cannot be excluded.

Limitations of the study are the cross-sectional nature of the associations studied. Strengths are the large variety of herpesviruses evaluated and the widely validated surrogate markers of atherosclerosis studied.

In summary, in this extensive study of the *Herpesviridiae* infecting humans, our data suggest a relationship of high seropositivity to varicella-zoster virus and cytomegalovirus with subclinical atherosclerosis. The concomitant finding of elevated inflammation and hypercoagulability biomarkers accompanying herpesvirus infection in virologically suppressed patients suggests a possible involvement of these mechanisms as contributing pathogenic factors. Our results illustrate a plausible scenario where several potentially interrelated pathogens, and pathogens' immune and inflammatory responses coexist in HIV-infected patients with subclinical atherosclerosis.
